# Society Meetings

**Published:** 1898-01

**Authors:** 


### Society Meetings.

THE Roswell Park Medical Club, at its November meeting, was entertained with two papers by Dr. Irving M. Snow, one entitled Certified milk in Buffalo, and the other Insolation in babies.

THE Elmira Academy of Medicine held its regular meeting Wednesday evening, December 1, 1897, under the presidency of Dr. Frank W. Ross. Prof. Veranus A. Moore, professor of comparative pathology and bacteriology at the New York State Veterinary College, Cornell University, delivered a lecture on The nature and prevention of anthrax, with special reference to a recent outbreak near Elmira, N. Y., with specimens, cultures and virus.

THE Buffalo Sanatory Club will hold a meeting the third Thursday in January, when it will discuss the subject of disinfection. The discussion will be subdivided as follows: general disinfection, by Dr. Thomas B. Carpenter; chemistry of disinfection, by Prof. John A. Miller; bacteriological disinfection, by Dr. H. U. Williams or Dr. H. G. Matzinger; legal rights of property owners in regard to placards, disinfection of houses and the like, by Leroy Parker, Esq.

THE Medical Society of the County of Chautauqua held its semi-annual meeting at Jamestown, December 14, 1897, under the presidency of Dr. Morris N. Bemus. The following-named physicians were elected to membership: Drs. Mary Armstrong Jones and J. J. Mahoney, of Jamestown, and Drs. G. E. Smith snd Thomas E. Soules, of Cherry Creek. The scientific session was devoted to a discussion of acute peritonitis. In the evening Hon. F. W. Stephens addressed the society upon the subject of Expert evidence from a lawyer's viewpoint, after which a dinner was served, at which twenty-eight covers were laid.

The Medico-legal Society of New York held its annual meeting at the Hotel Marlborough, Wednesday, December 15th, at 6.30 P. M. Dinner was served at that hour, and the regular order of business was taken up at 9 o'clock as follows: (1) Gas poisoning. The paper of Prof. W. B. McVey on this subject and Prof. C. A. Doremus was further discussed by Prof. M. C. White, M. D., of Yale University; Prof. Geo. B. Miller, M. D., chemist of society, of Philadelphia, and others who were invited to speak on the subject. (2) Paper by Prof. William J. Herdman, M. D., LL. D., of the University of Michigan, on Alleged traumatic neurasthenia. (3) Paper by Prof. Thomas Fassett Keyes, M. D., of Chicago, on Criminality and degeneracy. (4) Annual reports of the officers—treasurer, bacteriologist, chemist, microscopist, pathologist and toxicologist. (5) Annual reports of the sections of the society. (6) Report of the tellers on annual election of officers.

---

The Buffalo Academy of Medicine held meetings during the month of December, 1897, as follows:

*Section on Surgery.*—Tuesday evening, December 7th, indefinitely postponed until further notice on account of illness of essayists.

*Section on Medicine.*—Tuesday evening, December 14th, program: Clinical report, Lavage of stomach, followed by peripheral neuritis (including optic neuritis) and a peculiar edema, by Dr. J. C. Clemesha. Discussion opened by Dr. Allen A. Jones. The Jew in medicine, by Dr. Julius Ullman.

*Section on Pathology.*—Tuesday evening, December 21st, program: Report of two cases of scurvy, giving account of blood examination, by Dr. A. L. Benedict. The bacillus icteroides (yellow fever); History of its discovery, report of personal observations as to biological and other characters, and exhibition of cultures and preparations, by Drs. Frank J. Thornbury and Maj. A. Herst Appel, U. S. A. Exhibition of specimens, by Dr. Marcell Hartwig and Dr. A. L. Benedict. Trichinosis equi, first recorded case; presentation of microscopical slides and photographs, by Dr. F. J. Thornbury.

*Section on Obstetrics and Gynecology.*—Tuesday evening, December 28th, program: Appendicitis in its relation to obstetrics, by Dr. Eugene A. Smith.

The Medical Society of the County of Erie will hold its seventy-seventh annual meeting Tuesday, January 11, 1898, at Palace Arcade, observing the following program :

9.30 A. M. Business. President's annual address, Dr. Lucien Howe, Buffalo. Election of officers. Scientific contributions : Colle's fracture, Dr. J. G. Thompson, Angola ; Infant mortality, Dr. F. C. Gram, Buffalo ; reports of interesting cases.

12.30 P. M. Recess. X-ray examinations, Dr. Frederick Preiss, Buffalo. During recess Dr. Preiss will give opportunity for the examination with the X-rays of any cases presented by members of the society. As time will be limited, any member desiring to present a case for examination will please communicate with Dr. Preiss in advance of the meeting.

2.30 P. M. Some diarrheal diseases of children, Dr. F. H. Stanbro, Springville ; Formaldehyde disinfection, Dr. W. G. Bissell, Buffalo, bacteriologist to the health department; discussion by Drs. H. R. Hopkins, W. D. Greene, G. W. York and Jno. A. Miller.

3.30 P. M. Discussion on milk : Modified milk for infant feeding, G. E. Gordon, Esq., of Boston, Mass. ; followed by Drs. Irving M. Snow, Eugene Smith, Edward Clark, Ernest Wende, Dewitt Sherman and H. M. Hill. Reports of cases.

Members are invited to present brief reports of any cases of special interest observed in their practice.

